# Recent Technological Advances and Strategies for Arbovirus Vector Control

**DOI:** 10.3390/tropicalmed7090204

**Published:** 2022-08-23

**Authors:** Leonardo B. Koerich, Mauricio R. V. Sant’Anna, Ralph Huits

**Affiliations:** 1 Department of Parasitology, Universidade Federal de Minas Gerais, Belo Horizonte 31270-901, Brazil; 2 Department of Infectious Tropical Diseases and Microbiology, Ospedale Sacro Cuore Don Calabria, 37024 Negrar, Italy

In recent decades, we have seen the emergence and re-emergence of many arthropod-transmitted viruses (arboviruses) that pose important public health challenges worldwide [1]. Globally, dengue is the most widespread of these arboviruses with an estimated 3.9 billion people at risk of infection, particularly in tropical and subtropical areas.

In countries where climatic, social, and economic conditions favor the proliferation of vector mosquitoes, there are more than 500 recognized arbovirus species across several virus families and genera, and approximately 134 of these can cause human disease. Mosquitoes are the vectors of the most prevalent human arboviral diseases (dengue fever, chikungunya virus, Zika virus, yellow fever, Japanese encephalitis, and West Nile virus), causing more than 700,000 deaths annually [2]. Despite global efforts to control arbovirus vectors, arthropods continue to be a formidable public health adversary for endemic countries. The fight against arbovirus vectors is complicated and requires multiple factors: insect behavior and biology; dimensions of urban, rural, and sylvatic vector populations; the precariousness of urban sanitation; and habitat expansion through globalization and travel. Anthropization is a major foe in that equation, tipping the balance towards the spread of vector-borne human diseases.

In March 2022, the World Health Organization launched the Global Arbovirus Initiative [3], with the aim to build an integrated strategic plan to tackle emerging and re-emerging arboviruses with epidemic and pandemic potential, focusing on risk monitoring and detection, pandemic prevention, and quick response, building a coalition of partners. Recent advances in scientific technologies (e.g., genome sequencing, transcriptomics, and CRISPR-cas9 genome editing) increased our understanding of arbovirus vector biology. It has been 15 years since full *Aedes aegypti* genome sequencing [4], heralding the era of arbovirus vector genomics. The analysis of the *A. aegypti* genome and advances in population genomics have provided invaluable information about the origin of *A. aegypti* in West Africa, and its subsequent worldwide spread via the Americas to Asia, in the last 5 centuries [5]. Regarding arthropod–virus interactions, transcriptomes have allowed the identification of a wide variety of arthropod coding genes (mostly immune-related) and noncoding RNAs that present altered expression patterns upon virus infection. The development of metagenomics also sheds light on how Zika viruses can modulate gut bacteria in *A. aegypti*, suggesting that the gut microbiome is relevant to a successful arboviral arthropod infection [6]. 

The knowledge gained by the development of new technologies has facilitated the development of new strategies for vector control, beyond insecticide spraying. Successful initiatives such as the World Mosquito Program aim to replace wild-type *A. aegypti* populations with *A. aegypti* mosquitoes carrying *Wolbachia* endosymbionts, which have a reduced vector capacity for a variety of arboviruses such as dengue, Zika, chikungunya, Mayaro, and yellow fever. Clinical studies from Indonesia and Brazil have shown a reduction of ~70% of dengue cases in areas where the Wolbachia-transfected mosquitos were released [7,8]. 

Tropical Medicine and Infectious Disease is launching a Special Edition with a collection of papers on Emerging Topics in Arbovirus Vectors. This collection will deliver an exceptional compilation of recent advances in research and strategies to control arthropod vectors of viruses of global health concern. Furthermore, this Special Issue will provide scientists with up-to-date information on vector physiology, vector–virus and vector–host interactions, vector genomics, metagenomics, transcriptomics, population genetics/genomics, vector resistance to insecticides, and new strategies for vector control (e.g., CRISPR-cas9 genome editing, RNAi, and *Wolbachia* infected mosquitoes).

## Data Availability

Not applicable.
